# Myositis ossificans mimicking sarcoma: a not so rare bioptic diagnostic pitfall

**DOI:** 10.1186/s13052-020-00874-9

**Published:** 2020-07-31

**Authors:** Luisa Cortellazzo Wiel, Matteo Trevisan, Flora Maria Murru, Marco Rabusin, Egidio Barbi

**Affiliations:** 1 Department of Medicine, Surgery, and Health Sciences, University of Trieste, Piazzale Europa 1, 34127 Trieste, Italy; 2 Institute for Maternal and Child Health, IRCCS “Burlo Garofolo”, Trieste, Italy

**Keywords:** Myositis ossificans, Heterotopic calcification, Osteogenic sarcoma, Zonal pattern organisation, Case report

## Abstract

**Background:**

Myositis ossificans (MO) is a heterotopic bone formation in soft tissues, usually caused by traumas or neuropathies. Although the aetiology remains unclear, MO is supposed to be an osteoblast metaplasia with a benign and self-limiting course. Remarkably, at onset MO can be clinically, radiologically and histologically indistinguishable to soft tissue malignancies, especially in cases lacking a history of trauma, leading to misdiagnoses and improper treatments.

**Case presentation:**

A 13-year-old male was referred to the Oncology Department because of a previous diagnosis of osteogenic sarcoma of his left thigh. The diagnosis was made upon a history of isolated thigh pain in the absence of traumas, the evidence of a contrast-enhanced soft tissue mass on magnetic resonance imaging and the histological findings of atypical nuclei and mitotic figures. The lesion was eventually radiologically unchanged after five cycles of chemotherapy; thus, the child was referred for radical surgery. At admission, endorsing the child well-appearance, together with the evidence of a reduced calcified lesion on a further magnetic resonance, a clinical suspicion of myositis ossificans was raised. Hence, the excisional biopsy confirmed the pathognomonic zonal pattern of myositis ossificans.

**Conclusions:**

This case highlights some frequent diagnostic pitfalls facing myositis ossificans. A lacking history of traumas, along with a too early radiological and histological evaluation can lead to a misdiagnosis of soft tissue malignancies. Even in the absence of a clear history of trauma, a painful soft tissue swelling with a benign clinical course should raise the suspicion of myositis ossificans.

## Background

Myositis ossificans (MO) consists of the formation of lamellar bone in the context of soft tissues, especially large skeletal muscles of arms and thighs [1, 2]. Two different forms of acquired MO can be recognized: neurogenic and non-neurogenic; the latter can be divided, in turn, into post-traumatic circumscribed MO (60–75% of cases) [3] and idiopathic/pseudomalignant. Post-traumatic MO can result from both severe direct injuries and recurrent minor trauma, even in the form of abuse [4].

While pathogenesis is still not completely understood, the current hypothesis is that of an endothelial-mesenchymal transition, in which mesenchymal stem cells differentiate into chondrocytes and osteoblasts guided by a cytokine cascade following trauma, ischemia or inflammation [5].

The natural history of MO consists of a rapid overgrowth in the first 4 weeks, when osteoblasts and chondrocytes produce new osteoid matrix in the middle of the lesion. The typical peripheral calcifications are detectable between the fourth and tenth week when the lesion stops growing. Once the lesion is mature, the so-called “zonal pattern organization” can be radiologically and histologically appreciated, consisting on a central area of proliferating fibroblasts with possible necrosis and haemorrhage, followed by an intermediate zone of immature osteoid tissue along with cartilage, resulting from enchondral ossification and an outer shell of lamellar mature bone [6, 7].

The clinical presentation consists of painful swelling of the involved site with reduced range of motion of the adjacent joint [8]. A pointed anamnesis allows a prompt diagnosis in the majority of cases. Nevertheless, in the presence of a growing mass without any history of trauma, the suspicion of a bone or soft tissue cancer has to be raised. Even though radiological imaging can help identify the centripetal calcifications of the lesion, sparing the cortical bone, in the first weeks these features can lack. The biopsy is deserved to indeterminate lesions, but if performed too early or within the core lesion, the presence of pleomorphic osteoblasts with atypical nuclei and mitosis can be misleading. Hence, in the first weeks, MO can be almost indistinguishable from cancers [9]. A case of myositis ossificans through the common diagnostic pitfalls is reported.

## Case presentation

**Fig. 1:**
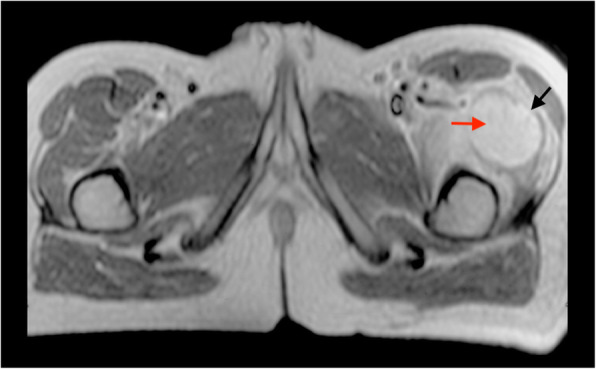
Contrast T1-weighted axial MRI: Well-defined contrast-enhanced lesion (5 × 4 × 3 cm) within the proximal third of the left vastus lateralis (red arrow) sparing the cortical bone and separated from surrounding oedema by a hypointense rim (black arrow)

**Fig. 2:**
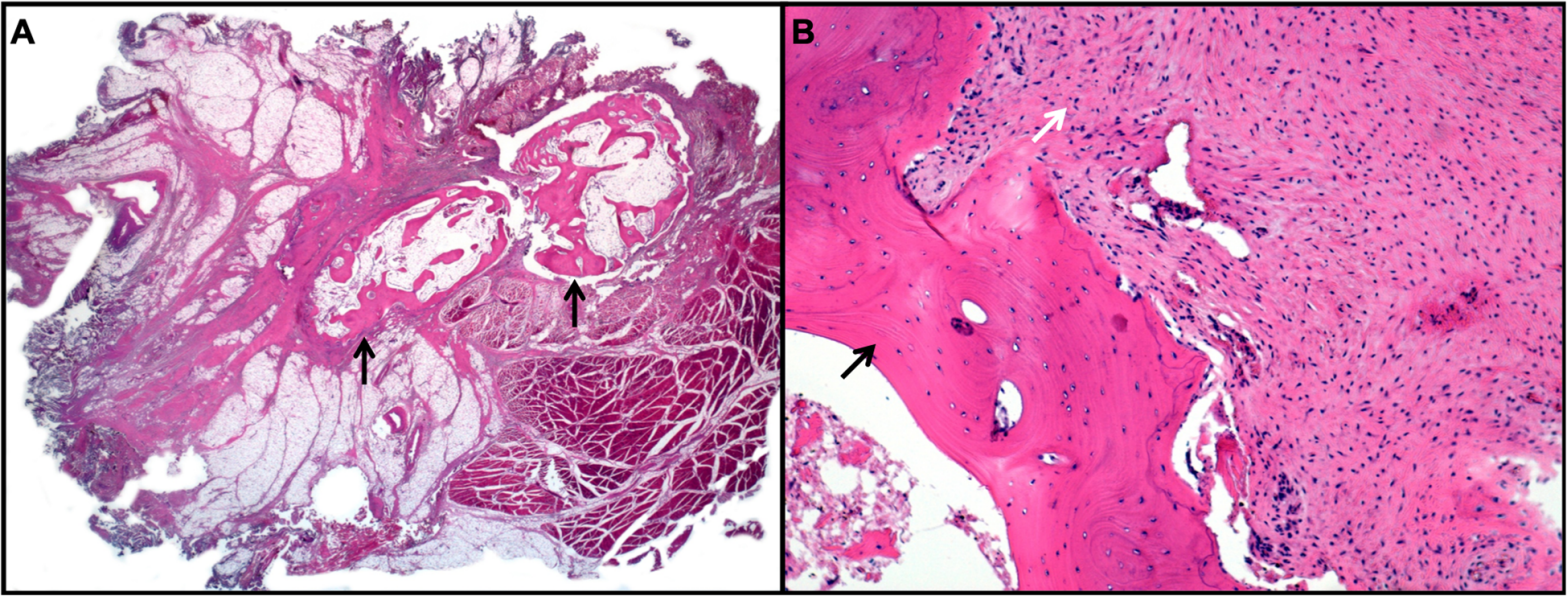
Histological sample from the thigh lesion. Panel **a**: hematoxylin and eosin (2X), mature lamellar bone within a stroma of adipo-muscular and striated muscular tissue. Panel **b**: hematoxylin and eosin (20X), absence of atypical mitosis or cellular pleomorphism within both bone (black arrow) and stromal tissue (white arrow)

A 13-year-old male was referred from another hospital to the Oncology Department after receiving a diagnosis of osteogenic sarcoma of his left thigh. Seven months before, he had started to complain about an isolated pain on his left thigh, in the absence of limp, fever or any history of trauma. After an unremarkable X-ray, he underwent a magnetic resonance imaging (MRI) of the left leg, showing a well-demarcated soft tissue mass of 5 × 4 × 3 cm within the proximal third of the left quadriceps, sparing the cortical bone (Fig. 1). The lesion enhanced homogenously by contrast and was surrounded by widespread oedema of the entire vastus lateralis and intermedius. A biopsy showed several large polygonal to spindle cells with atypical nuclei, mitotic figures and extensive necrosis, along with foci of abnormal osteoid formation, chondroid elements and calcifications. A diagnosis of osteogenic sarcoma was made. After five cycles of chemotherapy with methotrexate, adriamycin and cisplatin, the radiological findings were unchanged. The patient was therefore referred for surgery and further chemotherapy as needed. At admission, the clinical examination was unremarkable. An MRI confirmed the presence of a residual calcified muscle lesion of 4.2 × 1.2 × 0.7 cm. Endorsing the benign course of the disease, the clinical suspicion of myositis ossificans was raised. Hence, he underwent a sparing limb surgery. The histological sample showed the presence of three circumferential zones: islets of mature osteoblasts and fibroblasts without mitotic or atypical nuclei in the middle of the striated muscle, an interim zone with spindle cells surrounded by an osteoid stroma and a peripheral area with well-organized lamellar bones, confirming the diagnosis of myositis ossificans (Fig. 2). Neoplastic markers tested negative. The patient was discharged with the recommendation of muscular rehabilitation.

## Discussion

Even if MO generally displays the typical radiological and histological features in the course of the disease, it can represent a diagnostic challenge during the first weeks from onset, requiring a differential diagnosis with malignancy.

Plain radiographs typically show a lesion made up of a central radiolucent area indicating immature bone formation with a calcified peripheral rim of mature ossification. A thin radiolucent cleft separates the ossified mass from the adjacent cortex, which is intact, thus guiding the differential diagnosis with bone malignancies [10]. However, soft tissue calcifications are usually detectable not earlier than 4 weeks after the onset of the disease [11] and the lesion usually reaches its typical appearance after 6 months. Before this interval, MO can be radiologically indistinguishable to extraskeletal osteosarcomas. MRI allows an earlier recognition of the lesion: during the acute phase of the disease, it shows the presence of heterogeneous signal intensity on T1-weighted sequences without contrast enhancement, representative of blood products. These lesions will eventually progress to a pattern of lamellar bone with low signal intensity on all sequences [12]. To confirm MRI findings, the performance of computed tomography (CT) is recommended to recognise the characteristic centripetal calcifications [13] before they become detectable by standard radiography [14]. The main differential diagnoses of MO are soft tissue abscess and sarcoma: the latter typically displays contrast enhancement and generally lacks the peripheral calcified rim of MO [15].

Biopsy is deserved to indeterminate lesions. Remarkably through the sole performance of a fine needle aspiration cytology, as well as if the biopsy sample is taken from the lesion core or is performed too early, it is likely to run into the misdiagnosis of soft tissue sarcoma, due to the presence of isolated mitotic fibroblast-like cells [16].

Table 1 summarizes the main clinical, radiological and histological differences between MO and osteogenic sarcomas.

**Table 1:** Main differences between myositis ossificans and osteogenic sarcoma

	MYOSITIS OSSIFICANS	OSTEOGENIC SARCOMA
**CLINICAL**	Rapidly-growing, painful swelling and joint stiffness; History of trauma.	Local pain, swelling and limp; Night-time awakenings with bony pain; Pathological fractures;
**RADIOLOGICAL**	**Rx/CT**: calcified peripherical rim with a radiolucent cleft between the lesion and the cortical bone.	**Rx/CT**: Periosteal reaction, Codman’s triangle, sunburst sign; lobulated mass (cauliflower-like).
	**MRI**: early T2-weighted hyperintensity (oedema) and later hypointense rim in all sequences; Usually no contrast-enhancement.	**MRI**: heterogeneous or solid contrast-enhancement.
**HISTOLOGICAL**	“zonal pattern organization”: 1. Peripherical mature lamellar bone; 2. Middle zone: immature osteoid matrix; 3. Inner zone: proliferating fibroblast tissue.	Spindle/polygonal, malignant mesenchymal cells; hemorrhagic and necrotic lesions; MDM2 and CDK4 +.

*Abbreviations*: *CT* computed tomography, *MRI* magnetic resonance imaging

In this case, at the onset of disease, the absence of calcifications on radiological imaging and the finding of mitotic and atypical cells on the first biopsy led to a misdiagnosis of osteogenic sarcoma and the corresponding chemotherapy. However, the first MRI already exhibited some atypical features for osteosarcoma, consisting in the sharp demarcation of the soft-tissue mass, the presence of a circumscribing hypointense rim and oedema and the sparing of the cortical bone (Table 1). At referral, the repeated MRI showed a lesion not reduced in size after chemotherapy, without contrast-enhancement and with new calcifications. Finally, the excisional biopsy demonstrated the so-called “zonal pattern organization” allowing the diagnosis of MO.

Myositis ossificans is a benign self-limiting condition, and the treatment of choice is conservative. After an initial period of rest, gradual remobilization is recommended. Surgery is deserved to persistently symptomatic cases [17] and is preferably delayed until the complete maturation of the lesion has been reached, and ossification has stopped to prevent the occurrence of relapses [18, 19].

## Conclusion

This case highlights the diagnostic pitfalls of myositis ossificans. Chronic and aspecific symptoms without any history of trauma, together with a precocious radiological and histological evaluations can be misleading. When history, symptoms and imaging are not diagnostic, an excisional biopsy is recommended.

MO should be considered in the differential diagnosis of bone and soft tissue sarcomas in front of painful soft tissue swelling, valuing the radiological pattern and the benign course of the disease, even in the absence of a clear history of trauma, to avoid unnecessary treatments and to maximize functional outcomes.

## Data Availability

No supporting data are available.

## Abbreviations

MO: Myositis Ossificans
MRI: Magnetic Resonance Imaging
CT: Computed Tomography
